# Changes in peristalsis following per oral endoscopic myotomy in common esophageal motility disorders

**DOI:** 10.1007/s00464-025-12096-3

**Published:** 2025-08-28

**Authors:** Siddarth Vyas, Marc A. Ward, Brittany Buckmaster, Bola Aladegbami, Christine Wang, Gerald O. Ogola, Steven G. Leeds

**Affiliations:** 1 Department of Minimally Invasive Surgery, Baylor University Medical Center, 3500 Gaston Ave, Dallas, TX USA; 2 University of North Texas Health and Science Center, Fort Worth, TX USA; 3 Research Institute, Baylor Scott and White Health, Dallas, TX USA; 4 Center for Advanced Surgery, Baylor Scott and White Health, Dallas, TX USA; 5 Texas A&M College of Medicine, Bryan, TX USA

**Keywords:** Achalasia, Per-oral endoscopic myotomy, Peristalsis, High-resolution manometry, Motility, Jackhammer esophagus

## Abstract

**Background:**

Per-oral endoscopic myotomy has been used for a diagnosis of achalasia and non-achalasia motility disorders. Little is known about the manometric function of the esophageal body and LES after POEM. This study focuses on examination of the changes before and after POEM in the treatment of all 3 achalasia types as well as esophagogastric junction outflow obstruction and jackhammer esophagus.

**Methods:**

A prospectively maintained IRB approved database was retrospectively reviewed to find patients who were diagnosed with any type of achalasia, esophagogastric junction outflow obstruction, and jackhammer esophagus, and underwent POEM. Patients were included if they had pre-operative and post-operative high-resolution manometry. Primary endpoints were to identify changes in peristalsis as well as other manometric findings.

**Results:**

There were 265 patients who met the inclusion criteria. Type 1 achalasia showed the most change in restoration of peristalsis after POEM at 45.5% of patients. Type 2 achalasia improved peristalsis, but not significant from 46.8 to 60.3%. Type 3 achalasia patients lost some peristalsis from 82.5% of patient having peristalsis to 64.5% of patients after POEM. The DCI in these patients returned to a normal range after the procedure. Similar findings were seen in jackhammer esophagus where the DCI returned to a normal range. Esophagogastric junction outflow obstruction showed the biggest changes after POEM losing some peristalsis, but the DCI and wave amplitude significantly decreased (4993.2 to 902.5, 111 to 43.8, respectively).

**Conclusion:**

Per-oral endoscopic myotomy is used for treatment of all types of achalasia and non-achalasia motility disorders. Peristalsis is regained in some patients with Type 1 and 2 achalasia, where some peristalsis is lost in Type 3 achalasia. Type 3 and jackhammer esophagus have normal DCI after the procedure, and esophagogastric junction outflow obstruction shows the poorest outcomes with some patients losing peristalsis, decrease in DCI and wave amplitude.

## Introduction

Achalasia is the most common esophageal motility disorder characterized by dysphagia, regurgitation, and weight loss [1]. These symptoms are secondary to the loss of peristalsis in the esophageal body and failure of the lower esophageal sphincter (LES) to relax. Treatment has been focused on relieving the outflow obstruction to improve symptoms with an esophagomyotomy. Although the esophagomyotomy can be performed through a variety of techniques (endoscopic, laparoscopic, robotic, or open), POEM is becoming more commonly used given its precision and endoscopic approach [5, 6]. Because the esophagomyotomy has been so effective with achalasia, it has been used on non-achalasia esophageal motility disorders, such as esophagogastric junction outflow obstruction (EGJOO) and jackhammer esophagus [7].

The relationship of peristalsis and the outflow obstruction raises a question, does the outflow obstruction at the LES in achalasia patients eventually lead to aperistalsis of the esophageal body? Since achalasia involves aperistalsis of the esophageal body, many have questioned whether the idea of relieving the obstruction at the LES could lead to restoration of peristaltic activity in the esophagus. If this concept is true in achalasia, then the non-achalasia motility disorders may have a benefit too. A few studies have looked at encountering a post-POEM peristaltic wave, but its clinical significance has not been well studied. Hata et al. have examined over 600 patients to find that almost 20% had at least one discernable peristaltic wave [8]. Another study by Huh et al. demonstrated recovery of some peristalsis in 14 of 23 patients (61%) [9]. This study did include type 3 achalasia where it was noted that all 7 of the patients recovered peristalsis (100%). But between type 1 and 2 achalasia, it was 7 out of 16 patients (44%). Roman et al. showed that 57% of patients regained some contractility but included type 3 achalasia which had an 80% peristalsis rate post-POEM myotomy [10]. Shi et al. showed that symptoms did improve in the patients who had partial recovery of peristalsis based on Eckardt scores [11]. A total of 24 patients of their 103-patient cohort recovered some peristalsis (23%). Their Eckardt scores did not show a statistical significance, but the use of a GERD questionnaire showed a statistically lower incidence as well as a lower rate of post-POEM reflux esophagitis. To help fill in this knowledge gap, we set out to evaluate our patients undergoing POEM for achalasia and non-achalasia motility disorders and look at their manometric changes following POEM. The aim of this study is to see whether patients can regain peristalsis of the esophageal body following POEM and if these changes affect patient outcomes.

## Methods

We conducted a single-center, retrospective analysis of a prospectively maintained, Institutional Review Board-approved database of patients diagnosed with achalasia (any type), EGJOO, and jackhammer esophagus who underwent peroral endoscopic myotomy (POEM) between January 2015 and December 2023. Inclusion criteria included a diagnosis of achalasia, EGJOO, or jackhammer esophagus. Patients also needed to have availability of pre- and post-POEM high-resolution manometry (HRM). All analysis was done using Chicago Classification v4.0 [12]. Post-POEM HRM was performed at a median of 6 months following the procedure. This is a standardized follow-up protocol for these patients following the procedure. Patients were stratified based on their diagnosis and the changes in manometry findings before and after the procedure. HRM data performed with Medtronic (Medtronic, Minneapolis, Mn) equipment and software.

Primary outcomes included HRM peristalsis, DCI (distal contractile integral) in mmHg/cm/s, and distal wave amplitude in mmHg. Peristalsis restoration was defined as the presence of any non-zero, organized wave(s) on HRM. Secondary outcomes include Eckardt scores pre- and post-POEM that are grouped with patients who showed no peristalsis versus patients who showed improvement in peristalsis in Type 1 and 2 achalasia. These subtypes were chosen given the largest sample size, the more common subtypes, and given the Eckardt scoring system only applying to those with a diagnosis of achalasia.

### Pertinent POEM procedure details

It is planned for an anterior myotomy to extend 2cm beyond the high-pressure zone of the LES and generally 4–6cm proximal to it for types 1 and 2 achalasia, and EGJOO. For spastic disorders such as type 3 achalasia and jackhammer esophagus, the myotomy length was at least the distal 2/3 of the esophagus extending onto the stomach by at least 2cm. A dissecting cap is used and advanced to approximately 2–3 cm proximal to the projected start of the myotomy based on the prior measurement. A submucosal injection of diluted methylene blue solution is performed to raise a large bleb. The Hybrid knife (Erbe Medical, Tempe, Az) is used for tunnel creation. A selective myotomy is done transecting only circular muscle fibers. It is the decision of the surgeon to decide on the general length of the myotomy based on the motility disorder. Hemostasis is achieved and the mucosa is clipped closed with Resolution 360 hemoclips (Boston Scientific, Marlborough, Ma). Patients are typically discharged after the procedure with a full liquid diet for one week. Patients are not routinely started on antacids upon completion of the procedure at discharge.

### Statistical analysis

Continuous variables were summarized by mean and standard deviation for normal distributed variables, while median and interquartile range were used for non-normal distributed variables. Categorical variables were presented by the frequencies and percentages of events. Paired t tests were used to assess changes in HRM measurements from baseline to follow-up, while McNemar’s tests were used to assess association between indication of positive peristalsis at baseline and follow-up.

All statistical analyses were conducted with R version 4.0.3 statistical software, and all statistical tests were two-sided with a statistical significance level set at *p* values < 0.05.

## Results

At our institution, 328 patients underwent the POEM procedure in the time frame given. A total of 265 patients met the inclusion criteria. Table 1 shows the distribution of patients for each motility disorder and their demographics. Type 2 achalasia was the most diagnosed motility disorder with 44% of the patients. The majority of patients overall were female (59.9%), predominantly white (72%), and non-Hispanic (82%).

**Table 1:** Descriptive summary of patient characteristics by motility disorder type

	Type 1 (*N* = 58)	Type 2 (*N* = 116)	Type 3 (*N* = 57)	EGJOO (*N* = 25)	Jackhammer (*N* = 9)	Total (*N* = 266)
Age (years)—Mean (SD)	54.0 (18.0)	51.9 (19.3)	60.4 (13.5)	58.8 (13.4)	54.1 (13.8)	54.9 (17.5)
Sex						
Female	27 (54.0%)	55 (53.9%)	38 (69.1%)	18 (72.0%)	7 (77.8%)	144 (59.9%)
Male	23 (46.0%)	47 (46.1%)	17 (30.9%)	7 (28.0%)	2 (22.2%)	97 (40.1%)
Race						
White	38 (76.0%)	78 (76.5%)	49 (89.1%)	19 (76.0%)	6 (66.7%)	190 (78.9%)
Black	5 (10.0%)	12 (11.8%)	3 (5.5%)	4 (16.0%)	3 (33.3%)	27 (11.2%)
Other	7 (14.0%)	12 (11.8%)	3 (5.5%)	2 (8.0%)	0 (0.0%)	24 (9.9%)
Ethnicity						
Hispanic	4 (8.0%)	13 (12.7%)	5 (9.1%)	3 (12.0%)	0 (0.0%)	25 (10.3%)
Non-hispanic	46 (92.0%)	89 (87.3%)	50 (90.9%)	22 (88.0%)	9 (100.0%)	216 (89.7%)

*EGJOO* esophagogastric junction outflow obstruction; *SD* standard deviation

Post-procedure peristalsis and other manometric findings are seen in Table 2. Type 1 achalasia showed the most drastic difference in changes in peristalsis. Prior to the procedure, HRM identified 90.7% of the patients with no peristalsis, but following the procedure it decreased to 54.5% (*p* < 0.01). The mean basal LES pressure decreased from 34.7 to 17.3 (*p* < 0.01) and LES residual pressure also decreased significantly from 25.9 to 10.4 (*p* < 0.01). The DCI and wave amplitude did not have a significant change. Type 2 achalasia did not have a significant change in regaining peristalsis but it did improve after the procedure (46.8% to 60.3%, *p* = 0.21). There were 14.5% more of the patients with peristaltic activity after the procedure. In this cohort, mean wave amplitude was significantly better after the procedure (53.9 to 54.7, *p* = 0.01), but DCI did not improve (3863 to 2385, *p* = 0.21). Subtypes 1 and 2 were combined to show similar improvements as the Type 2 group.

**Table 2:** HRM outcomes for patients with Type 1 and 2 achalasia

Achalasia type	Outcome	Baseline	Follow-up	*p* value
Type 1	Positive peristalsis			< 0.01^1^
	No	49 (90.7%)	12 (54.5%)	
	Yes	5 (9.3%)	10 (45.5%)	
	LES basal mean			< 0.01^2^
	N	54	22	
	Mean (SD)	34.7 (20.2)	17.3 (9.8)	
	LES residual mean			< 0.01^2^
	N	54	22	
	Mean (SD)	25.9 (15.3)	10.4 (5.3)	
	DCI			0.97^2^
	N	4	8	
	Mean (SD)	1048.1 (1051.9)	1006.1 (1506.6)	
	Mean wave amplitude			0.99^2^
	N	4	8	
	Mean (SD)	42.2 (16.1)	46.8 (53.7)	
Type 2	Positive peristalsis			0.21^1^
	No	58 (53.2%)	23 (39.7%)	
	Yes	51 (46.8%)	35 (60.3%)	
	LES basal mean			< 0.01^2^
	N	106	58	
	Mean (SD)	50.1 (23.0)	23.4 (11.3)	
	LES residual mean			< 0.01^2^
	N	109	58	
	Mean (SD)	34.0 (14.6)	15.3 (8.5)	
	DCI			0.24^2^
	N	18	23	
	Mean (SD)	3863.3 (8777.2)	2385.7 (4150.9)	
	Mean wave amplitude			< 0.01^2^
	N	17	21	
	Mean (SD)	53.9 (30.6)	54.7 (33.2)	
Type 1 & 2	Positive peristalsis			
	No	107 (65.6%)	35 (43.8%)	< 0.01^1^
	Yes	56 (34.4%)	45 (56.2%)	
	LES basal mean			< 0.01^2^
	N	160	80	
	Mean (SD)	44.9 (23.2)	21.7 (11.2)	
	LES residual mean			< 0.01^2^
	N	163	80	
	Mean (SD)	31.3 (15.3)	14.0 (8.0)	
	DCI			0.22^2^
	N	22	31	
	Mean (SD)	3351.5 (7984.9)	2029.7 (3679.9)	
	Mean wave amplitude			< 0.01^2^
	N	21	29	
	Mean (SD)	51.6 (28.5)	52.5 (39.0)	

P value based on ^1^McNemar’s test; ^2^Paired *t* test

*HRM* high-resolution manometry; *LES* lower esophageal sphincter; *SD* standard deviation; *DCI* distal contractile integral

Table 3 shows the spastic disorders that included Type 3 achalasia and jackhammer esophagus. Type 3 achalasia showed that 82.5% of patients had some peristaltic activity prior to POEM, but after the procedure that number went down to 64.5% which was significant (*p* < 0.01). The DCI was drastically different going from mean of 20,774.8 to a normal range of 2234.2 (*p* < 0.01). Jackhammer esophagus had 100% peristalsis pre-operative and no patients lost peristalsis after the procedure. Once again, DCI did improve from 9,290.5 to a normal range of 1664 (*p* = 0.01).

**Table 3:** HRM outcomes for patients with Type 3 and Jackhammer achalasia

Achalasia type	Outcome	Baseline	Follow-up	*p* value
Type 3	Positive peristalsis			< 0.01^1^
	No	10 (17.5%)	11 (35.5%)	
	Yes	47 (82.5%)	20 (64.5%)	
	LES basal mean			< 0.01^2^
	N	55	31	
	Mean (SD)	50.3 (23.1)	26.2 (16.6)	
	LES residual mean			< 0.01^2^
	N	57	31	
	Mean (SD)	38.1 (56.2)	14.6 (11.0)	
	DCI			0.47^2^
	N	41	16	
	Mean (SD)	20,774.8 (95,388.3)	2234.2 (2479.2)	
	Mean wave amplitude			< 0.01^2^
	N	34	15	
	Mean (SD)	127.2 (72.3)	63.8 (36.5)	
Jackhammer	Positive peristalsis			< 0.01^1^
	No	0 (0.0%)	0 (0.0%)	
	Yes	8 (100.0%)	7 (100.0%)	
	LES basal mean			< 0.01^2^
	N	9	7	
	Mean (SD)	37.6 (17.6)	26.2 (8.9)	
	LES residual mean			0.18^2^
	N	9	7	
	Mean (SD)	13.3 (12.9)	15.1 (6.3)	
	DCI			< 0.01^2^
	N	6	4	
	Mean (SD)	9290.6 (4500.5)	1664.0 (1306.7)	
	Mean wave amplitude			< 0.01^2^
	N	4	3	
	Mean (SD)	192.6 (67.0)	31.6 (12.4)	
Type 3 & Jackhammer	Positive peristalsis			< 0.01^1^
	No	10 (15.4%)	11 (28.9%)	
	Yes	55 (84.6%)	27 (71.1%)	
	LES basal mean			< 0.01^2^
	N	64	38	
	Mean (SD)	48.5 (22.8)	26.2 (15.3)	
	LES residual mean			< 0.01^2^
	N	66	38	
	Mean (SD)	34.7 (53.1)	14.7 (10.2)	
	DCI			0.36^2^
	N	47	20	
	Mean (SD)	19,308.8 (89,046.7)	2120.2 (2275.3)	
	Mean wave amplitude			< 0.01^2^
	N	38	18	
	Mean (SD)	134.1 (73.8)	58.4 (35.6)	

P value based on ^1^McNemar’s test; ^2^Paired t test

*HRM* high-resolution manometry; *LES* lower esophageal sphincter; *SD* standard deviation; *DCI* distal contractile integral

Table 4 shows the last comparison was of EGJOO that showed 100% peristalsis pre-POEM and that number decreased to 83.3% after the procedure indicating 16.7% of patients lost peristalsis. DCI and wave amplitude both decreased. DCI went from 4993.2 to 902.5 (*p* < 0.01) and wave amplitude of 111 to 43.8 (*p* = 0.01).

**Table 4:** HRM outcomes for patients with EGJOO achalasia

Achalasia type	Outcome	Baseline	Follow-up	*p* value
EGJOO	Positive peristalsis			< 0.01^1^
	No	0 (0.0%)	3 (16.7%)	
	Yes	24 (100.0%)	15 (83.3%)	
	LES basal mean			< 0.01^2^
	N	25	18	
	Mean (SD)	53.8 (24.0)	24.5 (13.1)	
	LES residual mean			< 0.01^2^
	N	25	18	
	Mean (SD)	29.5 (12.4)	13.2 (5.9)	
	DCI			< 0.01^2^
	N	23	11	
	Mean (SD)	4993.2 (4174.7)	902.5 (662.8)	
	Mean wave amplitude			< 0.01^2^
	N	20	10	
	Mean (SD)	111.0 (67.9)	43.8 (29.6)	

P value based on ^1^McNemar’s test; ^2^Paired t test

*HRM* high-resolution manometry; *EGJOO* esophagogastric junction outflow obstruction; *LES* lower esophageal sphincter; *SD* standard deviation; *DCI* distal contractile integral

**Fig. 1:**
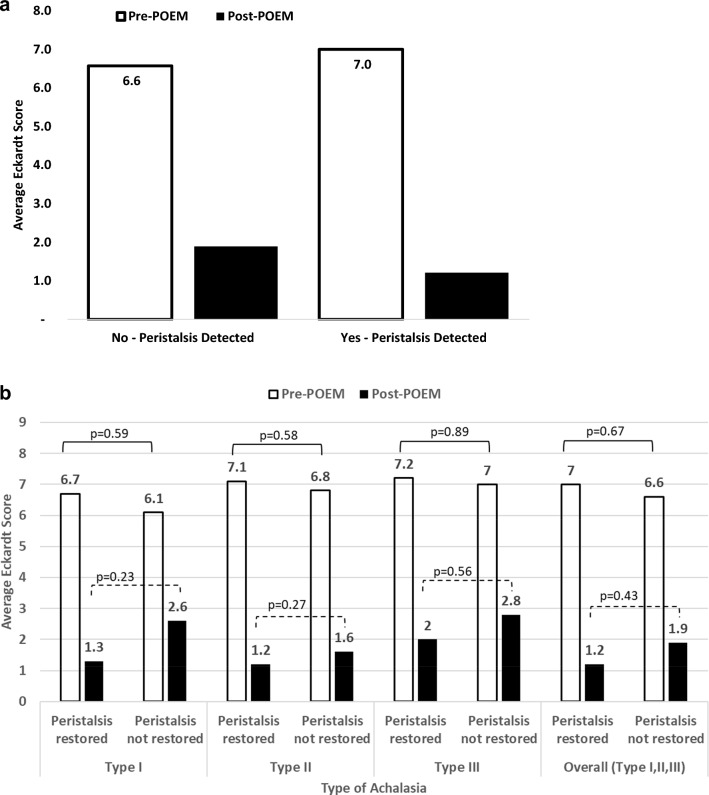
**a** Average Eckardt score by peristalsis restoration status. **b** Average Eckardt score by type of achalasia and peristalsis restoration status

Figure 1a shows pre- and post-POEM Eckardt scores for all types of achalasia categorized by patients who had no detectable peristalsis versus those patients who had some detectable peristalsis. It showed baseline Eckardt scores in the no peristalsis group of 6.6 and in the peristalsis group of 7.0. Post-POEM, these values dropped to 1.9 and 1.2, respectively (*p* = 0.43). Figure 1b shows the post-operative Eckardt scores for the different types of achalasia. This compares the Eckardt score differences for patients who showed some regain of peristalsis versus those who did not have peristalsis regained. There are improvements in Eckardt scores with regaining peristalsis in all three subtypes but none reached statistical significance.

**Fig. 2:**
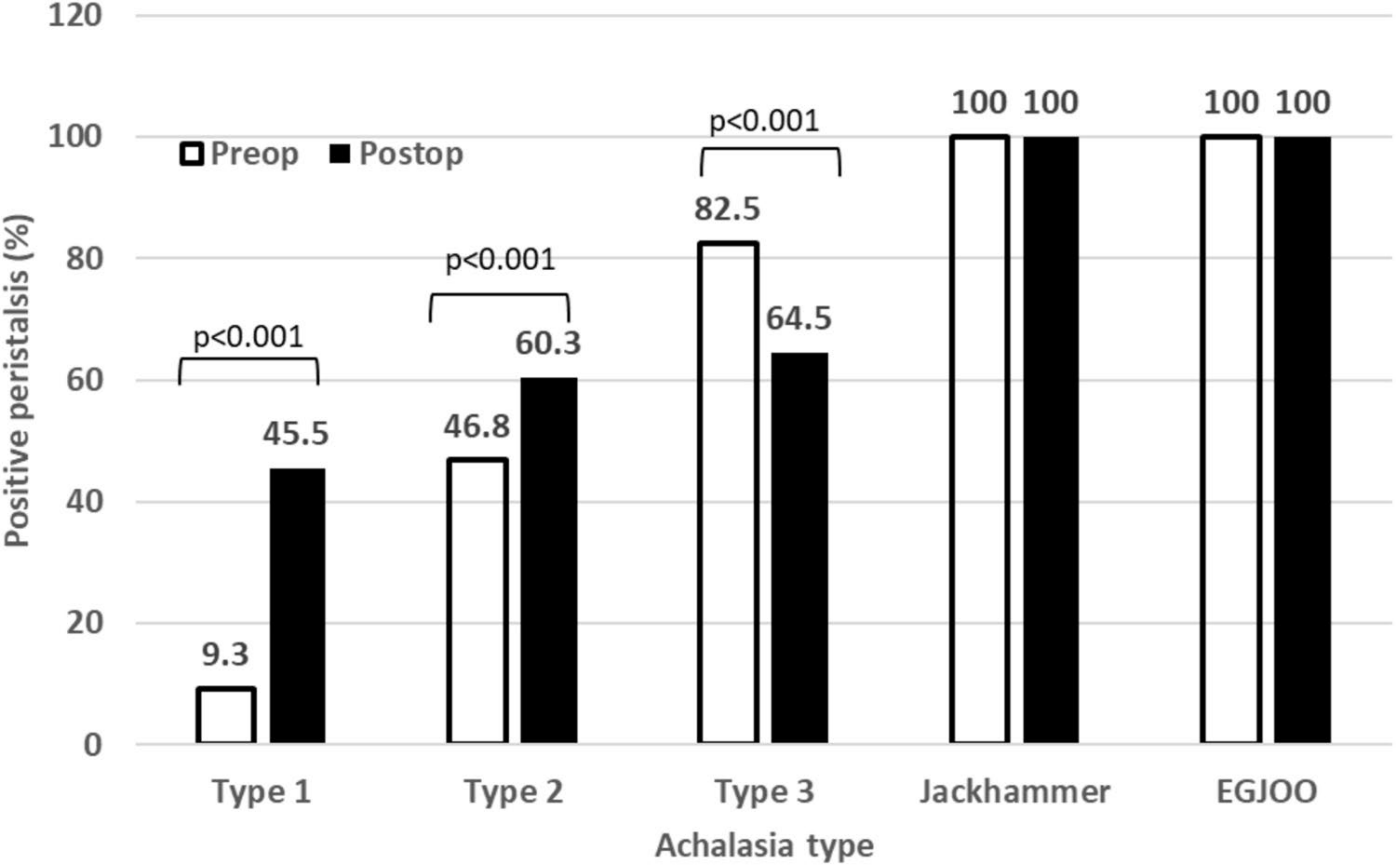
Percentage of patients with positive peristalsis by motility disorder type

**Fig. 3:**
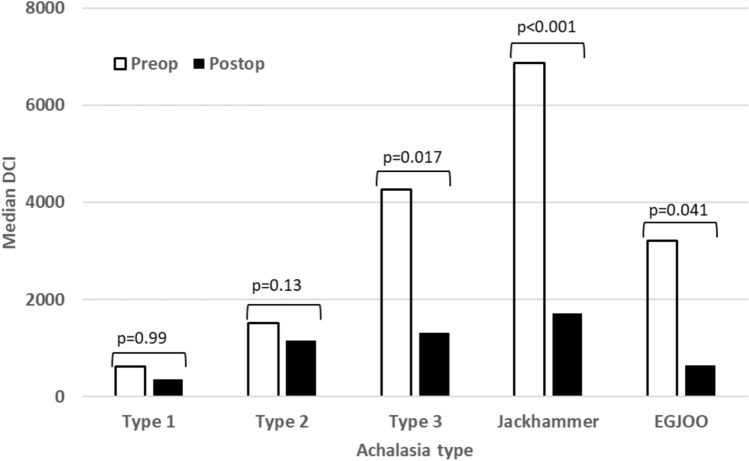
Median DCI by motility disorder type

Figure 2 shows the percent of patients with some peristalsis given each motility disorder after POEM procedure. The largest difference is Type 1 achalasia from 9.3 to 45.5 (*p* > 0.001). The expectation of jackhammer esophagus and EGJOO is that there would have been no change because peristalsis was present in all patients before POEM. Figure 3 shows the same difference between each motility disorder but with regard to DCI. All showed a significant change to worse DCI except from Type achalasia, but trended that way.

## Discussion

The POEM procedure has been a significant addition to management of esophageal motility disorders that include achalasia as well as non-achalasia motility disorders. It has been shown to improve post-operative symptoms, but the physiologic effect on the esophagus is not well studied. Here, we show the observation of esophageal body and LES activity following POEM in achalasia and non-achalasia motility disorders. This can give significant insight into predicting patient symptom outcomes and long-term effectiveness of POEM for these disorders.

The first interesting finding is that despite needing aperistalsis for a diagnosis of achalasia, the manometry software will encounter some activity it thinks is peristalsis. For example, the patients with Type 1 achalasia had far less esophageal motility than Type 2 achalasia prior to POEM to make the diagnosis of achalasia. This comparison was only 9.3% of patients with Type 1 achalasia had some peristalsis activity detected and nearly half of the Type 2 achalasia patients at 46.8%. In theory, the interpretation should show 0% peristalsis for all of these patients, but there is some activity measured. In Type 3 achalasia, nearly all the patients had some peristaltic activity at 82.5%. More appropriately, EGJOO and jackhammer had 100% peristalsis making the interpretation from the manometry software more dependable. This emphasizes the interpretation of an expert to recognize the correct disease diagnosis.

The second interesting finding is the DCI and peristalsis relationship in all of the patients. With the outflow obstruction alleviated with the myotomy by the POEM procedure, we expected the DCI improve as well as peristalsis. However, this was reflected in Type 1 and 2 achalasia, but not in type 3 achalasia. Appropriately, the DCI returned to a normal range (20,774.8 to 2,234.2), but the peristalsis suffered. The patients went from 82.5% of patients having peristalsis activity down to 64.5%, making the aperistalsis group increase from 17.5% to 35.5%. The explanation likely comes from the POEM procedure itself. In these patients, the average myotomy length is much longer than that of Type 1 and 2 achalasia which significantly affects its overall muscular tone. It decreases the vigor of the contraction, but the consequence is that some peristalsis may be lost. The effect on symptoms will need to be explored to see if these patients benefited from chest pain improvement but lost the improvement in dysphagia. If jackhammer esophagus is added to this analysis, the DCI appropriately drops to the normal range from 9,290.6 to 1664, but no patients lost peristalsis. This likely reinforces that jackhammer esophagus is a different physiologic change in the esophagus where Type 3 achalasia is a response to the outflow obstruction.

The third interesting finding is the effect POEM had on EGJOO patients. Three patients lost peristalsis which was surprising, but this was also reflected in the esophageal body findings. There was a significant drop in mean DCI from 4993.2 to 902.5 (*p* < 0.01). The mean wave amplitude reflected this drop also from 111 to 43.8 (*p* < 0.01). This means that patients with EGJOO who undergo a POEM procedure can have a chance to become aperistaltic with a decrease in contraction vigor with weakened wave amplitudes. It is our observation that patients diagnosed with EGJOO do not respond well with the POEM procedure, and this can be the explanation. Further examination of Eckardt scores for symptoms changes will help tease out the outcomes in this specific patient subset.

The fourth interesting finding is that the subjective questionnaires of Eckardt scoring pre- and post-POEM. The regain of peristalsis seemed to improve the Eckardt scores overall, but this did not reach significance. When divided into each subtype, a similar outcome was seen where the Eckardt scores are better but did not reach significance (Fig. 1b). We did see a significant amount of peristalsis improvement after the POEM procedure, but the DCI did suffer. Each motility disorder demonstrated a drastic drop in DCI which is likely the reasoning for the Eckardt scores not to be more significant. The pattern is that the outflow obstruction is relieved, the peristalsis improves, but the DCI suffers. This tells us a lot about the physiology of the esophagus but does not provide any bold predictions about patient subjective improvement post-POEM. Or, we may see a difference emerge with a different assessment tool or perhaps a larger sample size.

There are several limitations to this study. It has a retrospective nature and this can create some selection bias. Patients who are having more difficulty with symptoms after POEM procedure may be more likely to follow-up and allow for subsequent manometry procedure to be done possibly creating a selection bias. Also, the error on the manometry software that detects peristalsis in patients diagnosed with achalasia can be improved. If aperistalsis is a requirement for diagnosis of achalasia, then the software should be able to reflect that. Future studies should include the symptomology of patients after POEM to see if these manometric findings relate to the symptoms patients are experiencing. And finally, the finding of peristalsis regained after POEM and relieving the outflow obstruction is very important in the study of outcomes for patients. Longer follow-up in the patients with regained peristalsis versus those without any peristalsis should be studied in more depth.

In conclusion, this study focuses on the manometric findings before and after the POEM procedure. It was used for achalasia patients as well as the more common non-achalasia motility disorders. It showed that patients with Types 1 and 2 achalasia can regain peristalsis up to 22%. EGJOO seems to result in weaker esophageal body function following the POEM procedure. Spastic disorders such as Type 3 achalasia and Jackhammer esophagus have the DCI return to normal ranges, but can lose some peristaltic activity. Overall, this can provide insight into predicting outcomes after the POEM procedure and offering patient education on expectations. Additionally, it does provide insight into the motility disorders and the role the outflow obstruction plays in esophageal physiology.
